# Supplementary material for: The interactome of intact mitochondria by cross-linking mass spectrometry provides evidence for coexisting respiratory supercomplexes
Source: Mol Cell Proteomics. 2017 Dec 8;17(2):216–32. doi: 10.1074/mcp.RA117.000470 (PMC5795388; doi:10.1074/mcp.RA117.000470)
Supplement: Supplemental Data [file supp_RA117.000470_133922_0_supp_23978_fzffwf.zip › spectra_annotation/mito_DR_spectra_annotation/2-2-19-1-10-1.pdf]

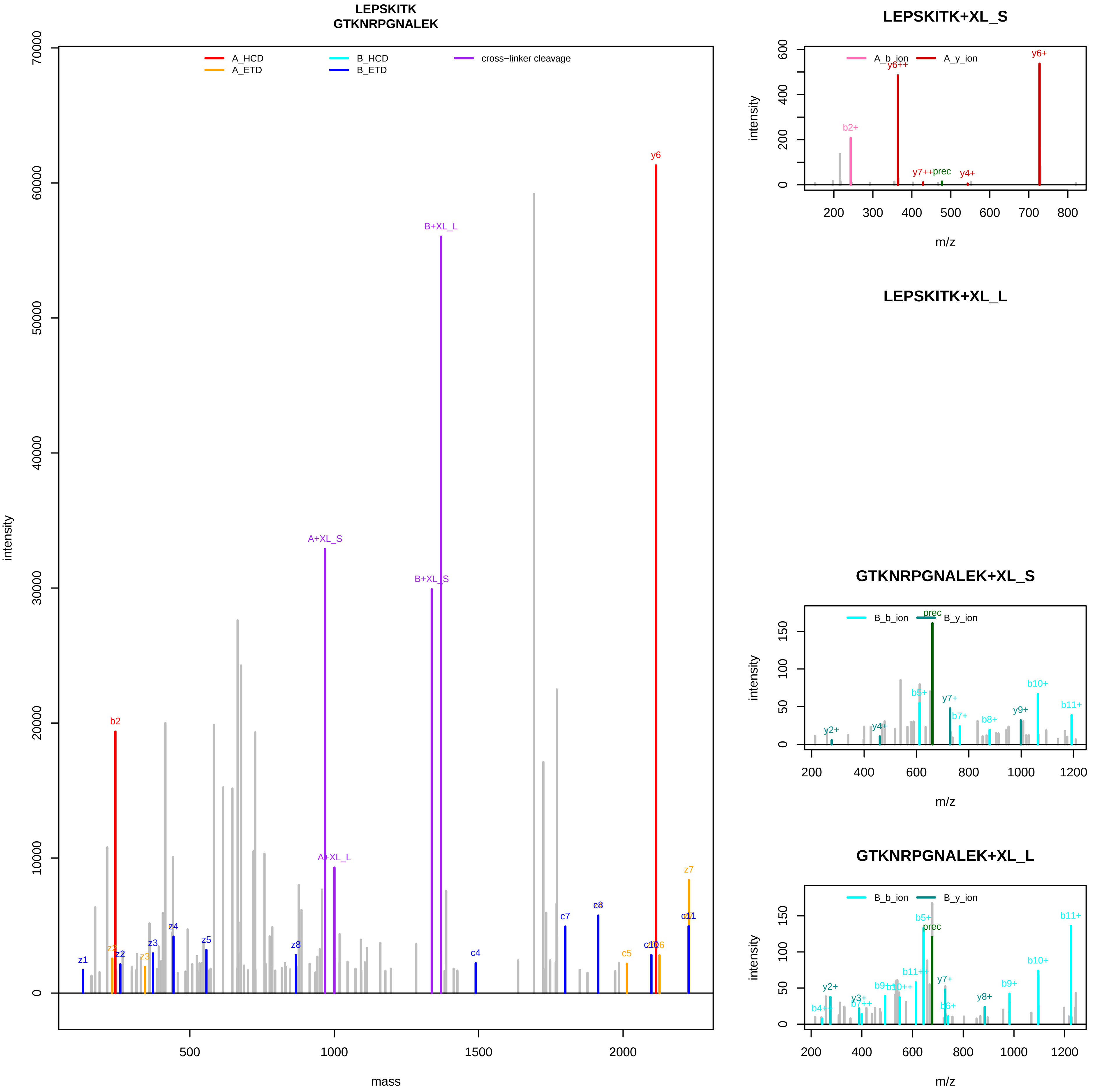
LEPSKITK
GTKNRPGNALEK
A_HCD
A_ETD
B_HCD
B_ETD
cross−linker cleavage
intensity
mass
0
10000
20000
30000
40000
50000
60000
70000
500
1000
1500
2000
b2
y6
z1
z2
z2
z3
z3
z4
z5
z8
A+XL_S
A+XL_L
B+XL_L
B+XL_S
c4
c7
c8
c5
c10
z7
c11
LEPSKITK+XL_S
A_b_ion
A_y_ion
b2+
y6++
y6+
y7++
prec
y4+
m/z
LEPSKITK+XL_L
GTKNRPGNALEK+XL_S
B_b_ion
B_y_ion
prec
y2+
y4+
b5+
y7+
b7+
b8+
y9+
b10+
b11+
GTKNRPGNALEK+XL_L
b4+
y2+
y3+
b7++
b9+
b10++
b11++
b5+
prec
y7+
b6+
y8+
b9+
b10+
b11+
m/z
